# Depth‐Resolved Localization Microangiography in the NIR‐II Window

**DOI:** 10.1002/advs.202204782

**Published:** 2022-11-20

**Authors:** Quanyu Zhou, Daniil Nozdriukhin, Zhenyue Chen, Lukas Glandorf, Urs A. T. Hofmann, Michael Reiss, Lin Tang, Xosé Luís Deán‐Ben, Daniel Razansky

**Affiliations:** ^1^ Institute of Pharmacology and Toxicology and Institute for Biomedical Engineering Faculty of Medicine University of Zurich Zurich 8057 Switzerland; ^2^ Institute for Biomedical Engineering Department of Information Technology and Electrical Engineering ETH Zurich Zurich 8093 Switzerland

**Keywords:** fluorescence microscopy, localization imaging, microangiography, second near‐infrared spectrum, stereovision

## Abstract

Detailed characterization of microvascular alterations requires high‐resolution 3D imaging methods capable of providing both morphological and functional information. Existing optical microscopy tools are routinely used for microangiography, yet offer suboptimal trade‐offs between the achievable field of view and spatial resolution with the intense light scattering in biological tissues further limiting the achievable penetration depth. Herein, a new approach for volumetric deep‐tissue microangiography based on stereovision combined with super‐resolution localization imaging is introduced that overcomes the spatial resolution limits imposed by light diffusion and optical diffraction in wide‐field imaging configurations. The method capitalizes on localization and tracking of flowing fluorescent particles in the second near‐infrared window (NIR‐II, ≈1000–1700 nm), with the third (depth) dimension added by triangulation and stereo‐matching of images acquired with two short‐wave infrared cameras operating in a dual‐view mode. The 3D imaging capability enabled with the proposed method facilitates a detailed visualization of microvascular networks and an accurate blood flow quantification. Experiments performed in tissue‐mimicking phantoms demonstrate that high resolution is preserved up to a depth of 4 mm in a turbid medium. Transcranial microangiography of the entire murine cortex and penetrating vessels is further demonstrated at capillary level resolution.

## Graphical abstract

A new 3D microangiography method is proposed based on localization of flowing fluorescent particles with a stereovision system in the second near‐infrared window. It overcomes the spatial resolution limits imposed by light diffusion, thus facilitating a detailed transcranial visualization of microvascular networks and an accurate blood flow quantification across the entire murine cortex and penetrating vessels with capillary level resolution.

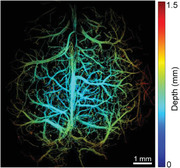

## 1 Introduction

Morphological and functional changes in the microcirculation are linked to the onset and progression of many pathologies.^[^ ^1^, ^2^ ^]^ Disease biomarkers can efficiently be derived by imaging‐based assessment of microvascular features, such as vessel size and density, perfusion, or blood flow velocity.^[^ ^3^ ^]^ Of particular importance is the microcirculation in the brain, which has been extensively studied in preclinical rodent models thus contributing to a better understanding of how oxygen and nutrients are transported into cells and how normal function is altered in brain tumor,^[^ ^4^, ^5^ ^]^ stroke,^[^ ^6^ ^]^ or Alzheimer's disease.^[^ ^7^, ^8^ ^]^ Multiple optical modalities are routinely used for angiographic imaging of the murine brain. Laser speckle contrast imaging (LSCI) is one of the most popular techniques for this purpose.^[^ ^9^ ^]^ It provides 2D dynamic blood perfusion maps from speckle pattern fluctuations induced by the motion of red blood cells. However, LSCI only covers a very shallow depth range, further hindering the quantification of blood flow velocity due to the equivocal speckle model.^[^ ^10^ ^]^ Multiphoton microscopy^[^ ^11^, ^12^ ^]^ was proposed as an alternative approach capitalizing on the optical sectioning capacity derived from its non‐linear excitation mechanism. Benefiting from confocality, 3D imaging could be achieved with several approaches such as mechanical objective translation along depth, focus‐tunable lenses,^[^ ^13^, ^14^ ^]^ remote focusing,^[^ ^15^ ^]^ or reverberation optical loops.^[^ ^16^ ^]^ Additionally, line scanning enables probing of flow velocities,^[^ ^17^ ^]^ but only in pre‐selected vessels within a small field of view (FOV), typically <1 mm. Optical coherence tomography has been used for microangiographic imaging at greater depths (≈1–2 mm) by detecting amplitude decorrelations between sequential B‐scans.^[^ ^18^ ^]^ It further enables quantifying blood flow velocity by exploiting the Doppler effect if a priori knowledge of the Doppler angle is available.^[^ ^19^ ^]^

The limited depth and FOV attainable with optical microscopy methods have fostered the development of new deep‐tissue imaging approaches based on ultrasound (US)^[^ ^20^ ^]^ and optoacoustics (OA).^[^ ^21^ ^]^ High‐resolution imaging at depths of several millimeters to centimeters is achieved with these methods by capitalizing on the insignificant scattering of US waves as compared to photons in soft biological tissues. US localization microscopy^[^ ^22^, ^23^ ^]^ has further enabled a striking resolution enhancement (≈tenfold) and simultaneous in‐plane velocity measurements via tracking of intravenously injected microbubbles. A similar approach can be implemented to mitigate resolution degradation due to scattering in wide‐field fluorescence imaging,^[^ ^24^, ^25^ ^]^ albeit only limited to planar (2D) representations. Volumetric (3D) microvascular imaging has been achieved with OA mesoscopy methods at depths of 2–3 mm.^[^ ^26^ ^]^ Localization optoacoustic tomography has further achieved super‐resolution 3D imaging of the mouse brain.^[^ ^27^, ^28^ ^]^ Advanced US and OA methods however require complex driving and acquisition electronics not easily accessible to biomedical researchers, while the need for acoustic coupling and acoustic aberrations induced by transcranial US propagation further compromise their performance.

The availability of efficient short‐wave infrared (SWIR) cameras capable of detecting photons in the 1000–1700 nm wavelength range opened a new avenue towards capturing ballistic photons in a second near‐infrared (NIR‐II) window with diminished light scattering and autofluorescence. Widefield,^[^ ^29^ ^]^ confocal,^[^ ^30^ ^]^ and light‐sheet microscopy^[^ ^31^, ^32^ ^]^ NIR‐II imaging significantly enhanced the imaging depth with respect to that achievable with other optical methods operating at visible or the first near‐infrared (NIR‐I) spectra. The development of NIR‐II contrast agent with high quantum efficiency further facilitated deep‐tissue imaging.^[^ ^29^, ^30^, ^33^, ^34^, ^35^ ^]^ Herein, we propose a new stereovision‐based volumetric deep‐tissue imaging approach in the NIR‐II window capable of achieving 3D transcranial super‐resolution microangiography of the mouse brain. The method is based on localization and tracking of flowing micro‐droplets encapsulating fluorescent quantum dots with two SWIR cameras providing widefield images in a dual‐view mode. Stereovision adds the missing depth information without the need for additional confocality or scanning in the axial (z) direction. The localization method effectively reduces the complicated feature matching processing to sparse dot matching, further attaining sub‐pixel resolution. We exploit the additional sparsity in the spatio‐temporal domain to minimize the ambiguity of registering dual‐view projections of densely distributed fluorescently‐labeled structures. The suggested approach is further shown to provide accurate quantification of the blood flow velocity, a key biomarker in many diseases.

## 2 Results

### 2.1 System Design and Characterization

**Figure 1:**
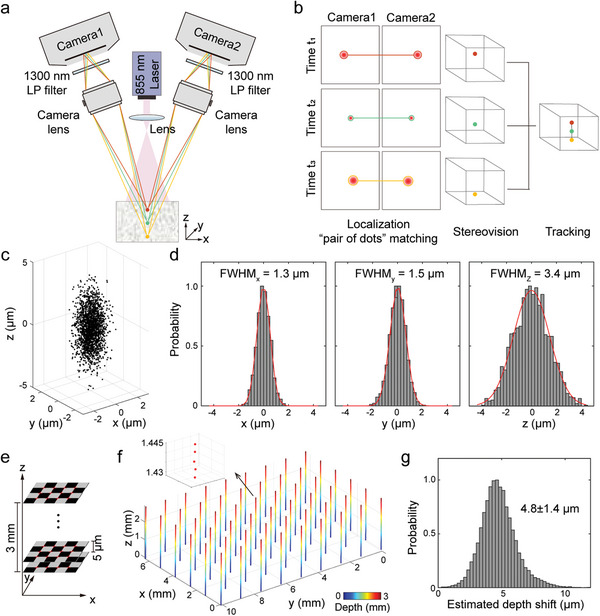
Concept of the depth‐resolved localization microangiography in the NIR‐II window. a) System lay‐out. The two cameras are positioned symmetrically with respect to the vertical axis with ±20° stereo angle. Fluorescence emitters located at different depths are projected to different pixels on the camera sensors. b) Localization and stereovision‐based image reconstruction workflow. c) 3D distribution of the localized positions corresponding to a single micro‐droplet for 2000 repetitive recordings. d) Histograms of the distribution of localized positions at the x, y, and z axes. Fitted Gaussian curves are shown along with the measured full‐width‐at‐half‐maxima (FWHM). e) Vertical scan of a printed checkerboard pattern along the z axis. f) Color‐coded 3D positions of the reconstructed crossing points of the pattern. Inset: zoom‐in view of the center point covering five layers. g) Histogram of the estimated depth shifts between adjacent layers. Mean and SD values are indicated.

Schematic representation of the proposed system is depicted in **Figure** **1**a (see Experimental Section for details). Epi‐illumination was provided by an 855 nm laser diode, while two identical SWIR cameras were used to collect the emitted fluorescence signals in dual‐view mode with a stereovision angle of ±20°. For this arrangement, fluorescence targets located at different depths are projected at different positions on the two sensors. Each pair of dots in these projections then enables 3D localization of isolated fluorescence emitters. Specifically, the lateral shift between the dots, the so‐called disparity, encodes the axial position of the emitter in the volume of interest (VOI), which is triangulated given the stereovision parameters (Figure 1b, see Experimental Section for details). The sparsity of flowing fluorescent micro‐droplets is exploited to localize their centers with sub‐pixel resolution in each of the images acquired with the cameras. This is followed by a search for matched pairs of dots located at similar horizontal coordinates after image rectification. A volumetric image is eventually rendered via superimposition of trajectories of flowing targets established by a 3D tracking algorithm (Figure 1b). Much like other localization‐based imaging methods, such as photo‐activated localization microscopy ^[^ ^36^ ^]^ or stochastic optical reconstruction microscopy ,^[^ ^37^ ^]^ the theoretical resolution of the proposed method is limited by the localization accuracy. The latter was estimated by rendering a dual‐view image stack of a static micro‐droplet on a flat microscopic slide at a 40 Hz frame rate for a total acquisition time of 50 s. The variability in the localized positions of the droplet serves to estimate the maximum achievable resolution (Figure 1c). Note that the size of the micro‐droplet is smaller than the diffraction limit of the optical system which is ≈27.7 µm in the current setting, that is, it can effectively be considered an ideal point source. Assuming the distribution of localized points is well‐approximated by a Gaussian function, the corresponding full‐width‐at‐half‐maxima (FWHM) are 1.3, 1.5, and 3.4 µm in x, y and z directions, respectively (Figure 1d). Note that the localization accuracy depends on the signal‐to‐noise ratio (SNR), which is higher for larger micro‐droplets. The system performance across the FOV was further validated by acquiring a bright‐field image stack of a flat checkerboard pattern, which was translated along the z axis with 5 µm steps over a 3 mm depth range using a motorized stage (Figure 1e). The crossing points between black and white squares were localized at each depth, plotted as a point cloud with color‐encoded depth in Figure 1f. The 5 µm depth difference between the scanned positions can be unambiguously distinguished in the enlarged view (inset in Figure 1f). The depth variance was encoded by the disparity of dual views (Figure S1a,b, Supporting Information). The histogram of estimated depth shifts between adjacent planes shown in Figure 1g has a mean and standard deviation (SD) of 4.8±1.4 µm, matching the scanning step. For an enhanced depth sensitivity, the stereovision angle needs to be expanded to increase the disparity between the dual views induced by a given shift of the sample in the depth direction (Figure S1c, Supporting Information). However, increasing the angle leads to a tilt between the two volumes defined by the depth‐of‐field of each camera, which results in a diminished overlap between the volumes provided by the dual views. To balance the depth sensitivity and effective imaging volume, a ±20° stereovision angle was thus chosen. These characterization measurements indicate that our method can in principle reach better spatial resolution than the capillary size, which corresponds to the smallest structures that can be imaged by particle tracking in the bloodstream.

### 2.2 3D Imaging Performance in Scattering Media

**Figure 2:**
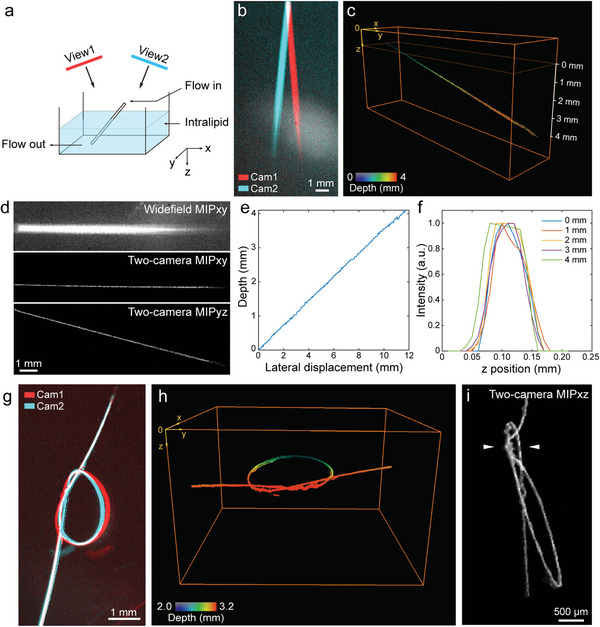
3D imaging performance in scattering media. a) Schematic illustration of a microtubing tilted inside a 1.2% Intralipid solution. b) Widefield‐equivalent images from the two cameras obtained by superimposing all the acquired frames during the injection of the micro‐droplet emulsion. c) Reconstructed 3D image of the tilted microtubing color‐coded for depth. d) Comparison between the widefield‐equivalent image from camera 1 and the maximum‐intensity‐projections (MIPs) of the reconstructed 3D volume based on stereovision. e) Change of calculated depth versus lateral displacement. f) Line profile along the axial direction at different depths labeled in (c). g) Widefield‐equivalent images from the two cameras of a tubing forming a knot pattern inside Intralipid solution. The images were obtained by superimposing all the acquired frames during the injection of micro‐droplets. h) Reconstructed 3D image of the knot pattern color‐coded for depth. i) MIP along the y axis of the image shown in (h). Overlapping segments of microtubing along z axis were indicated with white arrows.

The capability of our method to visualize structures within scattering media was first assessed in tissue‐mimicking phantoms. As an initial proof of principle, we imaged a microtubing immersed in a 1.2% Intralipid solution (**Figure** **2**a) and tilted such that the depth within the phantom increased with a constant gradient along the y axis. The concentration of Intralipid was chosen to simulate the reduced scattering coefficient in biological tissues.^[^ ^38^ ^]^ Volumetric imaging was performed by injecting micro‐droplets (diameter <20 µm) into the tubing at a constant flow rate (see Experimental Section for details). A dual‐view image stack was recorded for a total duration of 1 min at 18 Hz frame rate. The widefield‐equivalent images for each camera were obtained by superimposing all the recorded frames and overlaid together with cyan and red colors (Figure 2b). Along the depth gradient, the SNR and resolution in the widefield‐equivalent images rapidly deteriorated, while the displacement between dual views manifested the expected linear increase with depth. A volumetric view of the microtubing was reconstructed by localizing the 3D positions of individual flowing micro‐droplets via stereovision (Figure 2c). Comparison between the 2D widefield‐equivalent image from camera 1 and side views reconstructed with the localization method is shown in Figure 2d, illustrating that the latter clearly improves the achievable spatial resolution in a scattering medium. An approximately linear relationship between calculated depth and lateral displacement along the y axis was also observed, as expected (Figure 2e). Due to the fact that the phantom is immersed in aqueous solution, the calculated depth was rescaled by a factor of 1.33 to account for the refractive index mismatch between air and water. This was validated by comparing the calculated depth of the same tilted microtubing positioned in the intralipid solution and air (Figure S2, Supporting Information). Additionally, the line profile of the reconstructed microtubing at different depths was almost unaffected up to 4 mm depth in the Intralipid phantom (Figure 2f), corroborating the deep‐tissue imaging capability of the proposed system. We further evaluated the 3D performance of our method with a complex vascular‐shaped sample. Specifically, a tubing forming a knot pattern was immersed at ≈2 mm depth inside a 1.2% Intralipid solution. Dual‐view recording with the same parameters as the straight microtubing was performed following the injection of micro‐droplets. Widefield‐equivalent images from each camera, rendered by superimposing all the acquired frames, are shown in Figure 2g encoded with cyan and red colors. The 3D shape of the knot was clearly resolved in the reconstructed image (Figure 2h). In addition, the 239.4 µm depth difference of overlapping segments of the microtubing, matching well its outer diameter, could be distinguished from the side view (Figure 2i).

### 2.3 In Vivo Transcranial Mapping of Cerebral Microcirculation

**Figure 3:**
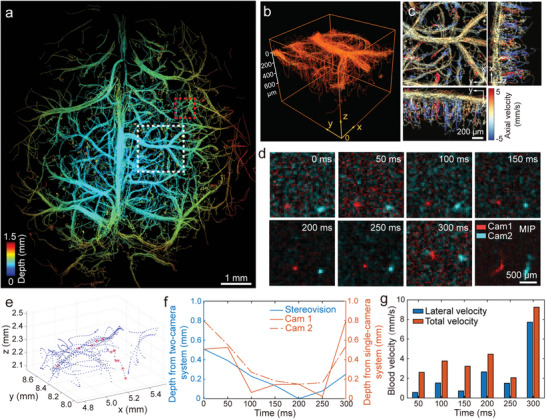
Transcranial volumetric microcirculation mapping of the mouse brain cortex. a) Reconstructed color‐coded microvascular depth map. b) Zoom‐in view of the volume of interest (VOI) indicated with a white square in (a). c) Maximum‐intensity‐projections (MIPs) along three views with color‐coded axial velocity. d) Representative time‐lapse images from the two cameras of a single micro‐droplet in the VOI indicated with a red square in (a). e) Reconstructed volumetric image. The trajectory of the micro‐droplet in (d) is labeled with red stars. f) Comparison of depth estimation with the proposed two‐camera system (stereovision‐based) versus single‐camera system (diffusion‐based estimation). g) Lateral and total velocity changes for the micro‐droplet in (d) over time.

We next demonstrated the capability of the proposed approach for transcranial imaging of murine cortical vasculature in vivo. For this, a dual‐view image stack was recorded at 40 Hz for 30 min during and post intravenous injection of the micro‐droplets. Owing to their small diameter of 4.73±2.25 µm (Figure S3, Supporting Information) below the reported capillary size,^[^ ^39^ ^]^ no significant capillary arrest was observed during the recordings (Video S1, Supporting Information). A depth‐resolved microcirculation map was acquired by tracing flowing micro‐droplets in 3D (**Figure** **3**a). Note that the observed depth gradient from the center to the edge of the image is due to the curvature of mouse brain (Figure 3a). Penetrating arterioles and venules at depths up to 600 µm are visible, as shown in the zoom‐in view of the selected VOI at approximately Bregma −1.5 mm (Figure 3b). Blood velocity maps of the same VOI from three views were also rendered via micro‐droplet tracking (Figure 3c). Note that the descending arterioles can only be distinguished from ascending venules in the axial velocity component. 3D imaging is essential for accurate quantification of blood flow velocity, which represents a powerful advantage of the proposed method over the previously reported implementation.^[^ ^25^ ^]^ Representative time‐lapse dual‐view images of a single micro‐droplet tracked through a penetrating vessel in a selected VOI are depicted in Figure 3d. At first, the separation distance between the two projections of the droplet in the two images has decreased and then slightly increased, which is in congruence with the observed depth changes in the localized 3D positions (Figure 3e). As cross‐validation, the depth of the same micro‐droplet was also estimated with a single‐camera by considering the dependence of the localized spot size on depth‐dependent light diffusion.^[^ ^25^ ^]^ While the depth estimates by our two‐camera system (stereovision‐based) and single‐camera system (diffusion‐based) were generally in good agreement (Figure 3f), larger fluctuations were observed in the measured depths with the single‐camera system, arguably due to the unknown and heterogeneous scattering properties of biological tissues. The depth resolving capacity enabled by our technique further provided more accurate estimates for the total micro‐droplet velocity based on 3D tracking, particularly in the penetrating vessels (Figure 3g). The fastest flow rate achievable with the proposed method was determined by the frame rate and “max‐linking‐distance” (MLD) parameter of the tracking algorithm. MLD defines the maximum displacement of a single micro‐droplet between consecutive frames allowed by the algorithm. It was set to 300 µm, which allowed for tracking particles flowing at up to 12 mm s^−1^ velocity when imaging with a 40 Hz frame rate. As shown in Figure 3g, micro‐droplet with a flow velocity of 9.25 mm s^−1^ can be successfully detected. According to the reported velocities in capillaries, venules, and arterioles in the murine brain under anesthesia,^[^ ^40^ ^]^ the suggested approach can be used to quantify the blood flow within a major portion of the cerebral vasculature.

## 3 Discussion and Conclusion

The proposed method capitalizes on the capability of localization‐based imaging to preserve high resolution in scattering media while further providing depth‐resolved information via triangulation and stereo matching of images captured from two angled views. The spatio‐temporal sparsity of the acquired image sequences of injected micro‐droplets lies at the core of the method's performance. On the one hand, adjacent fluorescence targets randomly distributed in the time domain facilitate the use of localization methods to achieve sub‐pixel resolution with both cameras. On the other hand, spatial sparsity in the imaged volume ensured that minimal structural overlaps occur in the dual‐view projections, thus eliminating ambiguities in sample matching. In this way, 3D high‐resolution fluorescence imaging at depths up to 4 mm was achieved in tissue‐mimicking phantoms. The method further allowed for transcranial visualization of penetrating microvessels across the entire murine cortex with capillary‐level resolution.

As compared to existing NIR‐II fluorescence imaging techniques, the proposed method offers several advantages related to the localization concept in stereovision. The reported dual‐camera system shares a similar FOV with other widefield systems,^[^ ^29^ ^]^ yet additionally benefits from the dual‐view detection to offer depth information. The implementation of localization‐based imaging further endows the dual‐camera system with high lateral and axial resolution beyond the diffraction‐limit of the objective, while significantly mitigating the scattering impact on spatial resolution (Figure 2d). The resolution degradation in scattering media has been largely mitigated by introducing confocality into the excitation/detection paths, for example, in confocal^[^ ^30^, ^41^ ^]^ and lightsheet^[^ ^31^, ^32^ ^]^ microscopy systems implemented in the NIR‐II window. However, these techniques are still restricted to microscopic (sub‐millimeter) FOV. Besides, depth information can only be achieved by time‐consuming mechanical scanning along the axial direction. Conversely, our system is capable of inferring the depth information across a much larger FOV while featuring a relatively simple optical design and well‐established camera calibration algorithms. Last but not least, 3D tracking of particle flow has provided full‐field functional information on blood flow velocity and direction, which is unattainable with conventional laser scanning microscopy.

Several technical advances are envisaged to boost the imaging performance envelope. First, the theoretical localization accuracy is ultimately determined by the SNR of the acquired images. Thereby, the spatial resolution can be improved by using SWIR cameras with better sensitivity in terms of quantum efficiency and noise level.^[^ ^42^ ^]^ An alternative way is to use contrast agents with a higher quantum yield to improve the brightness of single fluorescent particles. Note that SNR further determines the effective penetration depth. Second, a higher imaging rate could be attained by increasing the concentration of micro‐droplets in the injected emulsion and speeding up the acquisition frame rate of the cameras, since the amount of micro‐vessels being resolved generally scales with the number of frames. Yet, the micro‐droplets need to be distinguished individually by each camera and matched without ambiguity to attain accurate stereovision‐based 3D reconstruction. The frame rate of the two SWIR cameras used in this work was restricted to 50 Hz at a given SNR to synchronize both cameras and avoid frame loss with ≈5 ms required for real‐time data transfer in each exposure window due to lack of internal memory. Cameras with a higher frame rate can potentially increase the measurement speed by one order of magnitude.^[^ ^43^ ^]^ Third, the current camera calibration protocol is based on a “pinhole” camera model using printed calibration targets. The classical camera model is based on ray tracing in air without considering light refraction at the interfaces of different media. However, refractive index of biological tissues differs from air and can also exhibit significant heterogeneities. A camera model considering these refraction index mismatches can then improve the accuracy of the depth estimation. Finally, the potential application is not limited to angiographic imaging that relies on flowing fluorescence targets. Dynamic fluorescence changes in biological samples, for example, generated with calcium or voltage sensors due to neural activity, could be represented as “fingerprints” in the time domain.^[^ ^44^ ^]^ In this case, feature matching could potentially be performed by correlating temporal fluorescence fluctuations from dual views.

The newly introduced capabilities can be exploited in biomedical studies aiming at elucidating pathophysiological mechanisms of diseases. Cerebral vasculature is arranged in complex volumetric networks that can only be accurately characterized with 3D imaging methods,^[^ ^45^ ^]^ for instance in the context of ischemic and hemorrhagic strokes and selection of efficient therapeutic strategies.^[^ ^6^ ^]^ Quantitative readings of the blood flow performed transcranially over the entire murine cortex can greatly facilitate such studies. Microvascular function and blood flow are also impaired in Alzheimer's disease, particularly in cortical microvessels^[^ ^46^ ^]^ that can be identified with our method in murine models recapitulating pathological features in humans. The approach could also be used to assess angiogenesis, an important hallmark of cancer strongly correlating with tumor aggressiveness.^[^ ^47^ ^]^ Remodeling of small capillaries and arterioles further occurs in response to hypertension and type 2 diabetes mellitus, indicating an important role of the microcirculation in these and other diseases.^[^ ^48^ ^]^ Clinical translation of our approach would require the development and regulatory approval of nano‐ or micro‐particles suitable for human use. This can be facilitated by using particles encapsulating indocyanine green (ICG), a food and drug administration (FDA)‐approved agent that has been shown to exhibit a contrast in the NIR‐II window.^[^ ^43^ ^]^ Finally, our method can readily be combined with targeted molecular imaging approaches, for example, by employing tumor‐targeting contrast agents providing fluorescence contrast in the NIR‐II window.^[^ ^49^ ^]^

Taken together, the advantages provided by volumetric localization microangiography can greatly facilitate the characterization of microvascular morphology and circulation. The proposed technique enables the characterization of microvascular structures in vivo in a resolution‐depth range previously inaccessible with optical methods. Its relatively simple implementation is ideal for broad dissemination within the biomedical research community. The demonstrated performance to assess cortical microvessels is of particular relevance to investigating the underlying causes and effects of stroke and neurodegenerative diseases. In addition, the method can be used to advance our knowledge of the role of microcirculatory changes in cancer, diabetes, cardiovascular disorders, and other pathological conditions.

## 4 Experimental Section

### Experimental Setup

The layout of the proposed system followed a generalized widefield configuration adapted to provide stereovision performance by using two tilted cameras (Figure 1a). A continuous‐wave 855 nm laser diode was positioned between the two cameras to provide uniform epi‐illumination. Fluorescence responses were collected in dual‐view mode with the two cameras. Specifically, the two detection modules were fixed at ±20° symmetrically with respect to the main axis (vertical direction), each containing a 1300 nm long‐pass filter (FELH1300, Thorlabs, USA), a camera lens (LM50HCSW, 50 mm effective focal length, Kowa, Japan), and an InGaAs‐based SWIR camera (WiDy SenS 640V‐ST, NiT, France) mounted on an adjustable rotation stage (RP01/M, Thorlabs, USA). The magnification ratio of the detection system was set to ≈0.37. The pixel pitch on the camera sensor was 15 µm, corresponding to 40.5 µm in the object plane. For dual‐view recordings, the cameras were operated in a linear mode and synchronized with an external trigger signal. A non‐uniformity correction file was collected for each camera under specific exposure time and temperature, and subsequently applied to provide optimal image quality.

### Stereovision Calibration

Camera calibration of the proposed system was based on a “pinhole” camera model following the classical object‐based camera calibration pipeline, aiming at aligning the 3D world coordinate system of the object with the 2D coordinate system of each camera.^[^ ^50^, ^51^ ^]^ A printed checkerboard pattern, consisting of 5 × 6 squares (2 mm side length) with alternating white and black color, was attached to a microscopic slide as a planar surface for calibration. Twenty stereo image pairs recorded from different positions and orientations of the checkerboard pattern were imported to the stereo camera calibrator application^[^ ^50^, ^51^, ^52^, ^53^ ^]^ with Matlab (MathWorks, Matlab R2020a, USA) to calculate the stereovision parameters, including intrinsic parameters of each individual camera and translation/rotation between the two cameras. A two‐coefficient radial distortion model was chosen to minimize the mean reprojection error to 0.3 pixels.

### Micro‐Droplet Synthesis

Micro‐droplets were synthesized following the standard emulsification procedure. Oleic acid‐coated lead sulfide‐core‐type quantum dots (PbS QDs) in toluene with 1400 nm emission peak (747076, 10 mg mL^−1^, Sigma‐Aldrich, USA) were used in phantom experiments. First, the PbS QDs stock solution was precipitated in pure ethanol and redispersed in light mineral oil (M8410, Sigma‐Aldrich, USA) with a concentration of 50 mg mL^−1^. To check the potential fluorescence emission shift caused by the solvent change, the emission spectra of PbS QDs in toluene and light mineral oil were measured with NIR spectrofluorometer (FS5, Edinburgh Instruments Ltd., UK) under 800 nm excitation. Despite a blue‐shift of emission peak from 1321 (in toluene) to 1178 nm (in light mineral oil), the emitted fluorescence beyond 1300 nm could still be detected (Figure S4, Supporting Information). 50 µL PbS QDs solution was added to 0.8 mL double‐deionized (DI) water (resistivity of 18 MΩ cm, Millipore Milli‐Q A10) containing 3% v/v Tween20 surfactant (P1379, Sigma Aldrich, Germany). This was followed by vigorous shaking for 1 min using Vortex‐Genie 2 (Scientific Industries, USA). The resulting emulsion was filtered with a 20 µm cell strainer (43‐50020‐03, Pluriselect, Germany) and then inspected with a bright‐field microscope (Primostar 3, Carl Zeiss, Germany). System characterization and in vivo experiments were performed with core/shell lead sulfide/cadmium sulfide quantum dots (PbS/CdS QDs) in dichloromethane (DCM) with an emission maximum at 1600 nm (NBDY‐0038, 10 mg mL^−1^, Nirmidas Biotech, USA). The emulsification process remained the same but without pre‐concentration and phase‐changing steps. To characterize the size distribution of the micro‐droplets, widefield images were captured with a commercial bright‐field microscope (Figure S3, Supporting Information), followed by recognition of individual micro‐droplets and calculation of their diameter using build‐in functions (Threshold, Analyze particles) of ImageJ (https://imagej.nih.gov/ij/).

### System Characterization

To characterize the spatial resolution, micro‐droplets made of PbS/CdS QDs in DCM filtered by a 1 µm cell strainer (43‐50001‐13, Pluriselect, Germany) were diluted one hundred times. A single droplet was distributed on the microscopic slide with a cover slip. A sequence of images (image stack) of the micro‐droplet was recorded with both cameras at an exposure time of 20 ms to achieve the optimal SNR. In total, 2000 frames were recorded to provide a quantitative measurement of localization accuracy in 3D. The micro‐droplet remained static during the total acquisition duration. The 3D performance across the entire FOV was tested by imaging a printed checkerboard pattern (6 × 9 squares, 1.34 mm side length) in bright‐field mode under an infrared lamp (IL21, Beurer, Germany). A dual‐camera image stack of the checkerboard pattern was acquired at each z‐position controlled with a high‐accuracy motorized stage (SMV170‐13‐1, Standa, Lithuania) at a step size of 5 µm across a 3 mm depth range.

### 3D Image Reconstruction

The image processing workflow includes four steps, namely, localization, matching of the dot pairs, stereovision, and flow tracking (Figure 1b). First, for each single‐view image stack, an averaged image over the full acquisition time was subtracted from each frame to remove the static background resulting from autofluorescence and stray light. Subsequently, local intensity maxima were detected using the open‐source TrackNTrace toolbox.^[^ ^54^ ^]^ After dark current correction, the wavelet filtering plugin was used for candidate selection, followed by Gaussian‐fitting to extract centroids with sub‐pixel precision. The detection parameters were optimized for each camera once and kept constant for subsequent datasets. Note that each localized point was undistorted through employing the stereo parameters established with the camera calibration process to correct for lens distortion. Second, stereo images acquired at each time point were rectified to ensure that the corresponding points were located at similar horizontal coordinates. Thus, localized points from the same droplet (pairs of dots) were identified by simply comparing the difference between their y coordinates by setting a threshold, benefiting from the spatio‐temporal sparseness of flowing targets. In the case more than one matched point was recognized, the matched pairs were excluded from the subsequent reconstruction process since it was most likely caused by several micro‐droplets located in close proximity. Third, for each recognized pair of dots, the 3D world coordinates of the micro‐droplet were derived based on standard triangulation. Since each 2D point on the sensor represented a back‐projected ray determined by the intrinsic parameter of camera, triangulation was performed to find the intersection point of two rays from dual views with the given 2D camera coordinates and stereo parameters, thus rendering a point cloud in 3D space. Last, the trajectory of each localized microdroplet was tracked in 3D with the Simpletracker algorithm (https://github.com/tinevez/simpletracker) by identifying the closest sources in consecutive frames. The search range was defined as “max‐linking‐distance” (MLD) adjusted according to the frame rate, estimated flow velocity, and concentration of micro‐droplets. Furthermore, a threshold for the trajectory length was set to remove the potential false connections and stacked microdroplets. The lateral and axial velocity components of flowing droplets were calculated with the given frame rate and 3D displacement of individual micro‐droplets. The final (compounded) localization image was formed via superimposition of trajectories whereas the corresponding velocity map was calculated by averaging and then superimposing the velocities estimated in each voxel.

### Phantom Preparation

Two phantoms were prepared to validate the 3D performance of the proposed method in scattering media. The tip of an Eppendorf microloader (EP5242956003‐192EA, Sigma‐Aldrich, USA) was used as a microtubing (137 µm I.D., 231 µm O.D.) in phantom experiments. This microloader was connected to a syringe filled with a micro‐droplet emulsion composed of PbS QDs. The micro‐tubing was positioned tilted inside a tank filled with 1.2% Intralipid in DI water to mimic the averaged scattering properties of biological tissues. The speed of injection of micro‐droplets was adjusted in the range of 2 to 4 mm s^−1^ with a syringe pump (NE‐300, New Era Pump Systems, USA) to avoid motion artifacts caused by the limited frame rate of the camera. In addition, another microtubing was bent to form a “knot” pattern, fixed on a flat microscopic slide, and positioned at ≈2 mm depth below surface of the 1.2% Intralipid solution. The micro‐droplet emulsion was injected through the tubing with controlled velocity. For dual‐view recording, 50 ms exposure time, corresponding to 18 Hz frame rate, and a total acquisition time of 1 min were used for all phantom experiments.

### In Vivo Animal Experiment

An athymic nude‐Fox1nu mouse (female, 7‐week‐old, Envigo BMS B.V., Netherlands) was used for in vivo cerebrovascular imaging. The mouse was anesthetized with isofluorane inhalation (3% v/v for induction and 1.5% v/v for maintenance) with an oxygen/air (20%/80%) mixture on the heating pad. The scalp was removed after a subcutaneous injection of 0.1 mg kg^−1^ Buprenorphine (Temgesic, Indivior Schweiz AG, Switzerland) to suppress pain sensation, while the skull was kept intact. A dual‐view image stack was recorded for 30 min during and after intravenous injection of the micro‐droplet emulsion (1.23 × 10^6^ micro‐droplets mL^−1^). Exposure time of the SWIR cameras was set to 20 ms with a frame rate of 40 Hz. A time window of 5 ms between consecutive exposures was spared for real‐time data transfer. After image acquisition, the mouse was euthanized while still under anesthesia. All animal experiments were carried out in accordance with the Swiss Federal Act on Animal Protection and approved by the Cantonal Veterinary Office Zurich.

## Conflict of Interest

The authors declare no conflict of interest.

## Author Contributions

X.L.D.B. and D.R. conceived the experimental design. Q.Z. developed the experimental system. Q.Z. and D.N. carried out the experiments. Q.Z., Z.C., and L.G. conducted the data analysis and visualization. U.H. contributed to software development. M.R. contributed to the animal experiments. L.T. assisted with the spectral measurements. D.R. supervised the study. All authors contributed to writing and revising the manuscript.

## Supporting information

Supporting Information

Supporting Video 1

## Data Availability

The data that support the findings of this study are available from the corresponding author upon reasonable request.
