# Supplementary material for: Depth‐Resolved Localization Microangiography in the NIR‐II Window
Source: Adv Sci (Weinh). 2022 Nov 20;10(1):2204782. doi: 10.1002/advs.202204782 (PMC9811471; doi:10.1002/advs.202204782)
Supplement: Supplementary file 1 — Supporting Information [file ADVS-10-2204782-s001.pdf]

ADVANCED SCIENCE
Open Access

Supporting Information

for *Adv. Sci.*, DOI 10.1002/advs.202204782

Depth-Resolved Localization Microangiography in the NIR-II Window

Quanyu Zhou, Daniil Nozdriukhin, Zhenyue Chen, Lukas Glandorf, Urs A. T. Hofmann, Michael Reiss, Lin Tang, Xosé Luís Deán-Ben and Daniel Razansky**

Supporting Information for

Depth-resolved localization microangiography in the NIR-II window

Quanyu Zhou,[1,2] Daniil Nozdriukhin,[1,2] Zhenyue Chen,[1,2] Lukas Glandorf,[1,2]
Urs A.T. Hofmann,[1,2] Michael Reiss,[1,2] Lin Tang,[1,2] Xosé Luís Deán-Ben,[1,2,*] and Daniel Razansky[1,2,*]

[1]Institute of Pharmacology and Toxicology and Institute for Biomedical Engineering, Faculty of Medicine, University of Zurich, 8057 Zurich, Switzerland

[2]Institute for Biomedical Engineering, Department of Information Technology and Electrical Engineering, ETH Zurich, 8093 Zurich, Switzerland

*Correspondence

Daniel Razansky, Institute for Biomedical Engineering, Wolfgang-Pauli-Str. 27, 8093 Zurich, Switzerland

Email: daniel.razansky@uzh.ch

Xosé Luís Deán-Ben, Institute for Biomedical Engineering, Wolfgang-Pauli-Str. 27, 8093 Zurich, Switzerland

Email: xl.deanben@pharma.uzh.ch

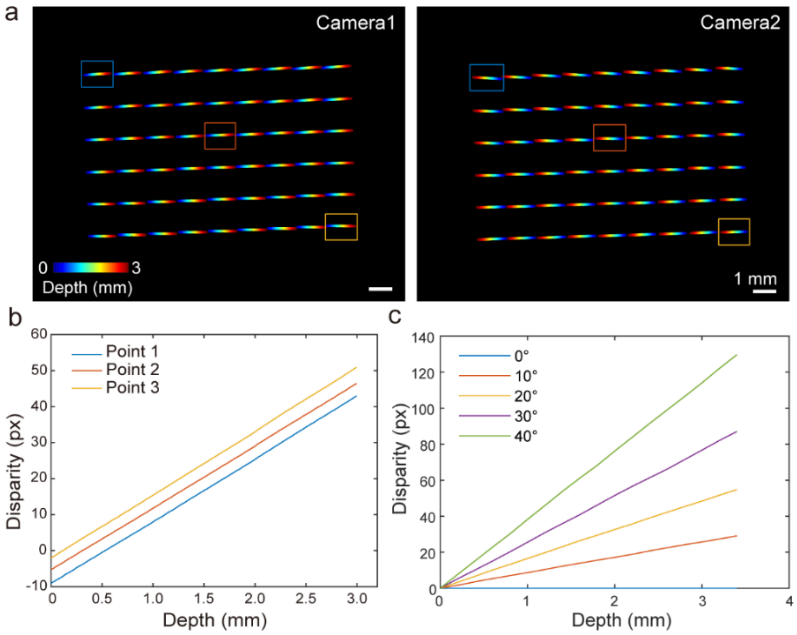

Figure S1 Projections of crossing points in the checkerboard pattern scanned along the axial (z) direction and relationship between the disparity and depth at different stereovision angles. (a) Localized crossing points on both cameras corresponding to different depths recorded with ±20° stereovision angle. **(b)** Calculated disparity (lateral shift between dual views) versus axial displacement of three selected points from the center and edge in the checkerboard pattern labeled in (a). **(c)** Calculated disparity versus axial displacement of the center point in the checkerboard pattern when the stereovision angle was changed from ±0° (disparity = 0, dual views converged to a single view) to ±40°.

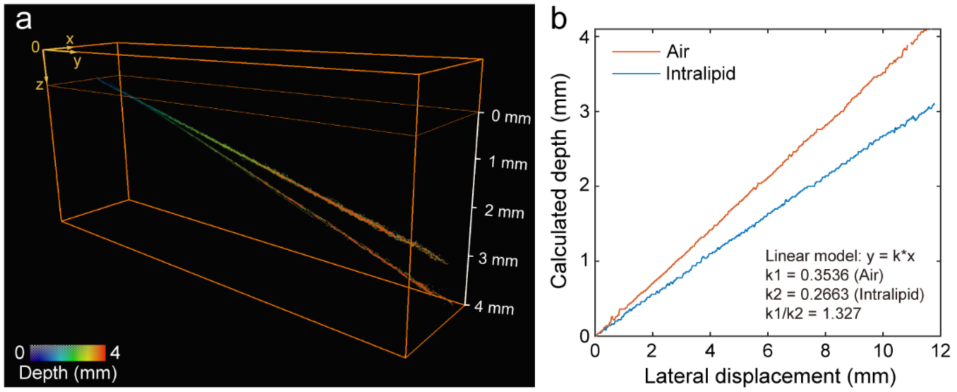

Figure S2. Influence of refractive index mismatch on depth estimation. (a) Reconstructed image of a tilted microtubing (with a constant depth gradient along y axis) positioned in the 1.2% Intralipid solution and air, respectively. **(b)** The calculated depth of the microtubing in both air and Intralipid shows a linear increase along y axis, as expected. The slope is reduced for the Intralipid case due to refractive index mismatch between air and water. After fitting a linear model, the ratio between the fitted slopes in both cases was 1.327, which is close to the refractive index of water (n = 1.33).

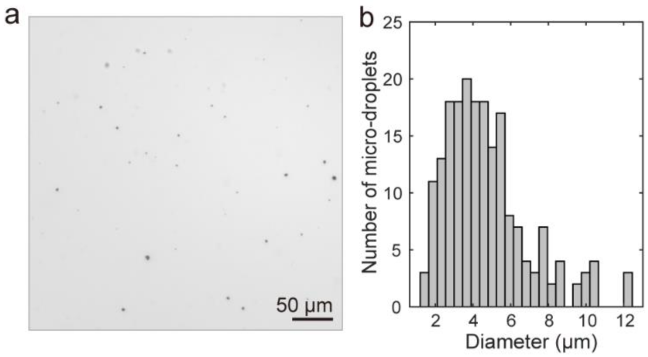

Figure S3. Representative bright-field image of micro-droplets made of core/shell lead sulfide/cadmium sulfide quantum dots (PbS/CdS QDs) in dichloromethane (DCM) and distribution of measured diameters. (a) Widefield image of micro-droplets distributed on the microscopic slide acquired with a bright-field microscope. **(b)** Histogram of micro-droplet diameter distribution with mean and standard deviation (SD) values equal to 4.73±2.25 µm. 197 micro-droplets were considered for diameter estimation.

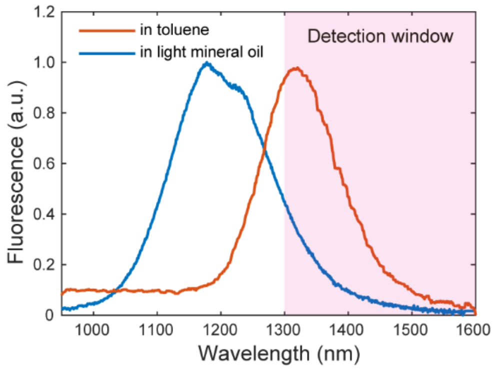

Figure S4. Emission spectra of lead sulfide-core-type quantum dots (PbS QDs) in toluene and light mineral oil (excited by 800 nm). A blue-shift of the emission peak in mineral oil was observed. The detection window corresponding to the 1300 nm long-pass filter used in this work is labeled in pink.
